# Cooperative Environment Scans Based on a Multi-Robot System

**DOI:** 10.3390/s150306483

**Published:** 2015-03-17

**Authors:** Ji-Wook Kwon

**Affiliations:** Yonsei Institute of Convergence Technology, Yonsei University, Songdogwahak-ro, Yeonsu-gu, Incheon 406-840, Korea; E-Mail: bluemichael@yonsei.ac.kr; Tel.: +82-32-749-5861; Fax: +82-32-818-5801

**Keywords:** cooperative environment scan system, multi-robot system, laser scanner, LRF, LiDAR

## Abstract

This paper proposes a cooperative environment scan system (CESS) using multiple robots, where each robot has low-cost range finders and low processing power. To organize and maintain the CESS, a base robot monitors the positions of the child robots, controls them, and builds a map of the unknown environment, while the child robots with low performance range finders provide obstacle information. Even though each child robot provides approximated and limited information of the obstacles, CESS replaces the single LRF, which has a high cost, because much of the information is acquired and accumulated by a number of the child robots. Moreover, the proposed CESS extends the measurement boundaries and detects obstacles hidden behind others. To show the performance of the proposed system and compare this with the numerical models of the commercialized 2D and 3D laser scanners, simulation results are included.

## 1. Introduction

The description of an unknown environment has received attention, as it can be utilized in path planning [1,2,3] and localization [4,5,6,7] in autonomous robot systems, which should be required to achieve safe and efficient movement. To acquire obstacle information for the description of an environment (this will be called a map), various range finders such as infrared, ultrasonic, vision, and laser sensors have been implemented on the autonomous robots.

To detect and describe the obstacles in an unknown environment, infrared and ultrasonic sensors have been implemented on robots for a long time, because they have provided good solutions in simple applications [8,9] due to their low cost and simple output data. However, since they provide only approximate information, a detailed map cannot be achieved. Vision systems such as time of flight (ToF) cameras have also been employed [10,11]. Since ToF cameras provide depth images that include distance information with large amounts of noise, autonomous robots can acquire obstacle information using the vision system. However, its detectable area is small, it can be difficult to extract obstacle information in image, and it cannot detect obstacles hidden behind other obstacles. To acquire precise obstacle information in a large area, LRF has been utilized such that the one-channel laser scanner for a 2D plane and a multi-channel laser scanner for a 3D space have been implemented on autonomous robots [12,13,14,15]. They provide detailed obstacle information as a point cloud with which to build the map. However, LRF has a high cost, and it still cannot provide invisible obstacle information. To overcome the detectable area limitation of LRF, simultaneously localization and mapping (SLAM) algorithms [4,5,6,7] and multi-robot exploration and mapping algorithms [16,17,18,19] have been proposed. It is possible that the map of a large environment can be built by estimating the position of the autonomous robots moving in the unknown environment. However, if loop-closing and map-merging algorithms that can correct integrated estimation errors are not guaranteed, the map-building procedure does not provide accurate environmental information. Since loop-closing derives the robot’s additional movement and map-merging procedures require a high processing burden, SLAM and multi-robot exploration and mapping algorithms increase the cost of the robotic system. In addition, in the case of multi-robot exploration and mapping, because all robots should implement LRFs, the cost of the entire system can increase as the number of robots increases.

Thus, this paper proposes a cooperative environment scanning sensor system (CESS) based on multiple robots that can replace a single LRF in an autonomous robot. In the proposed CESS, the multi-robot system is used as a single sensor. To the authors’ knowledge, a sensor system based on multiple robots instead of a single sensor device has not been previously proposed in the literature. In CESS, the multiple child robots provide obstacle information to the autonomous robot (which will be termed the base robot). The child robots provide obstacle information measured with low-cost range finders to the base robot, and the base robot controls the child robots. For an accurate map of the environment around the base robot, in CESS, it is important to know the position of the child robots. Thus, in this paper, two relative positioning systems are employed as follows: (a) visual sensor-based systems [19,20,21,22] and (b) ultra-wide band (UWB)-based system [23,24,25]. First, the vision-based positioning system [19,20,21,22] measures the position of the child robot using artificial markers, image target trackers, and projective geometry. When this positioning system is used, the child robots should move in the visible area, since the vision system of the base robot should see all child robots. Second, in the UWB-based positioning system, the child robots move within the boundaries of the UWB system, even though they are in invisible areas, because the UWB-based positioning system can detect objects hidden behind others. In addition, the proposed CESS presents both the 2D plane and 3D space according to the vertical angle of the range finders implemented on the child robot. When all the vertical angles of the range finders on the child robots are zero, CESS can describe the obstacle information on the plane (*i.e.*, CESS replaces 1-ch LRF); however, not all of their vertical angles are zero, so CESS can build a 3D obstacle map (*i.e.*, CESS replaces multi-channel LRF).

When CESS is employed in an autonomous robot (*i.e.*, base robot) in moving in an unknown environment instead of a single LRF, the following contributions are achieved. First, the organizing cost for the sensors scanning the environment is reduced. CESS can reduce the cost of the entire system by over 80% compared to LRF. Second, if the base robot implements a UWB-based positioning system detecting objects hidden behind others, CESS can acquire information about the obstacles behind others without a SLAM algorithm; while the performance of the range finders on the child robots is limited, these contributions are achieved due to the advantages of using a multi-robot system. In other words, even though the measurement of each child robot is not enough to build a precise map, the accuracy of the map increases and it describes more invisible information, since the base robot is provided much information by the child robots.

This paper is organized as follows: we describe the multiple robot system considered in the paper in Section 2. In Section 3, we propose control mechanisms for the child robots with respect to the positioning systems. To demonstrate the usefulness and performance of the proposed CESS, the simulation results are presented in Section 4. Finally, the conclusions of this study are given in Section 5.

## 2. Multiple Robot System for Detecting Obstacles

**Figure 1:**
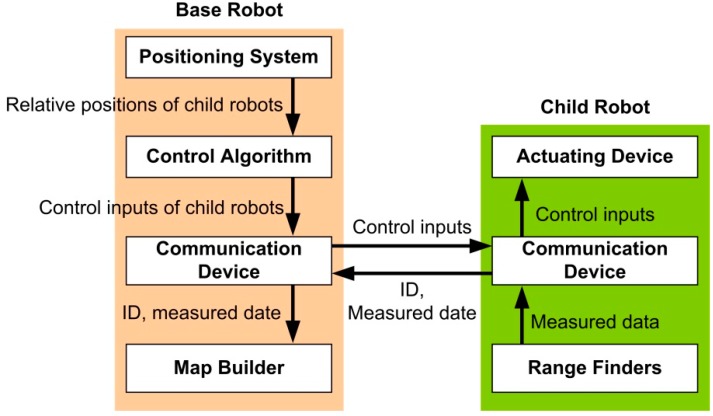
The CESS architecture.

To maintain CESS, the base robot controls all child robots via a wireless communication device and the child robots provide obstacle information around the base robot. To detail the cooperation between the base and child robots, Figure 1 shows the CESS architecture.

Figure 1 shows that CESS is a centralized system, because the base robot monitors the positions of all the child robots, controls them, acquires all obstacle information, and builds the map. First, the positioning system on the base robot provides the positions and orientations of the child robots for the control algorithm and the map builder. If artificial markers are used, it is possible that the ID, the relative position, and the orientation are measured [19,20,21]. However, if the artificial markers are not utilized, the child robot can be identified using the initial position, specified templates, and kinematic model in Equation (1), and the control inputs [22,26]. Second, since the child robot just provides obstacle information without the controller and its own positioning system, the child robot does not require a high-level processor (e.g., high performance MCU) or additional positioning sensors except the range finders and communication device. Finally, to estimate the position and control the child robot, it is assumed that the child robot is an underactuated system with non-holonomic constraint in Cartesian coordinates [27,28]. The kinematic model of the child robot is thus described as:

(1) $$\left[\begin{array}{c} {\dot{x}}_{i}\\ {\dot{y}}_{i}\\ {\dot{\theta }}_{i} \end{array}\right]=\left[\begin{array}{cc} \cos\theta_{i} & 0\\ \sin\theta_{i} & 0\\ 0 & 1 \end{array}\right]\left[\begin{array}{c} v_{i}\\ \omega_{i} \end{array}\right]$$

where (*x_i_*, *y_i_*) is the position of the *i_th_* child robot, *θ_i_* is the orientation, and *v_i_* and *ω_i_* are the linear and angular velocities, respectively, which will be designed as control inputs.

**Figure 2:**
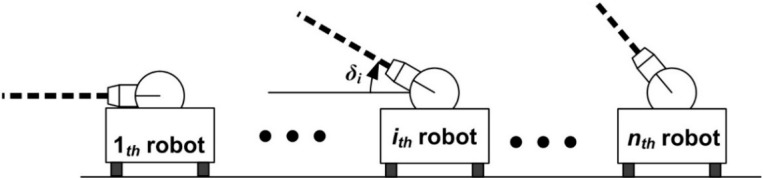
The vertical angles of the range finders on the child robots.

After the estimation of the position and control of the child robot, consider the measurements from the range finders on the child robot. As mentioned before, CESS presents both 2D and 3D information from the vertical angles of the range finders on the child robot, as depicted in Figure 2.

In Figure 2, *δ_i_* is the vertical angle of the range finder. In the case that all the vertical angles are 0 (*i.e.*, *δ*_1_ = *δ*_2_ = ··· = *δ_n_* = 0), a 2D plane is described. On the other hand, if the vertical angles of the sensors of all robots are not zero, CESS describes the 3D space information. Since the positions of the child robots are monitored by the base robot, the information of the detected obstacles is acquired as:

*x_p_* = *d*sin(*δ_i_*)cos(*θ_i_*)+*x_i_* *y_p_ = d*sin(*δ_i_*)sin(*θ_i_*)*+y_i_* *z_p_ = d*sin(*δ_i_*) (2)

where *d* is the measurement of the range finders. Note that the robots can be equipped with more than one range finder.

To connect the base and child robots and maintain CESS, the gateway communication model in [29] is employed. In this gateway model, the base robot serves as the gateway, and each child robot sends its measurements to the base robot and receives motion control inputs. For the gateway communication model, this paper assumes that there is little communication delay and noise. Note that since little information is transferred between the base and child robots, and the performance of commercialized communication devices such as Zigbee, Bluetooth, and Wi-Fi have improved, this assumption can be reliable. Of course, a communication time delay can occur as the number of child robots increases; thus, the number of the child robots should be modified with respect to the performance of communication devices.

## 3. Control Mechanisms According to the Positioning Systems

**Figure 3:**
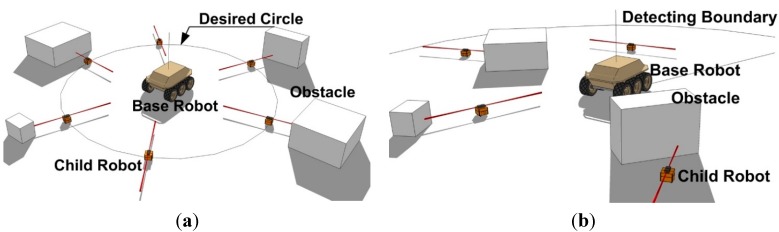
Two CESS control strategies with respect to the positioning systems. (**a**) CESS using the vector field based control law with vision based positioning system; (**b**) CESS using the behavior based control algorithm with the UWB-based positioning system.

The CESS coverage area is determined by the positioning system on the base robot. If a vision-based positioning system is utilized, child robots should move in the visible area of the base robot, on the other hand, if the UWB-based positioning system is implemented, the child robots move in the boundary of the positioning system without considering visibility. Accordingly, the control strategies are chosen by the positioning system as depicted in Figure 3.

As can be seen in Figure 3a, in the case of the vision-based positioning system, the child robots move in the desired circle designed in a visible area of the base robot, since the base robot can only monitor the visible child robots. To guarantee visibility, the vector field based multiple robot control algorithm in [27] is employed. However, if the UWB-based positioning system in Figure 3b is implemented, the child robots can move anywhere in the boundary of the positioning system even though they are invisible. Thus, in this case, the behavior based control scheme in [30,31] is adaptable.

**Remark** **1.** *To choose the positioning system, the vision- and UWB-based positioning systems are compared as follows. First, in the vision-based positioning system, all the child robots have artificial markers by which the relative position and orientation between the base and child robots are provided; however, the positioning system is limited in the visible area. Second, the UWB-based positioning system monitors the invisible child robots behind obstacles; however, it is difficult to identify the child robots. Therefore, the positioning system should be determined with respect to the desired specification of a given system*.

### 3.1. Vector Field Based Multiple Robot Control Algorithm for a Vision-Based Positioning System

**Figure 4:**
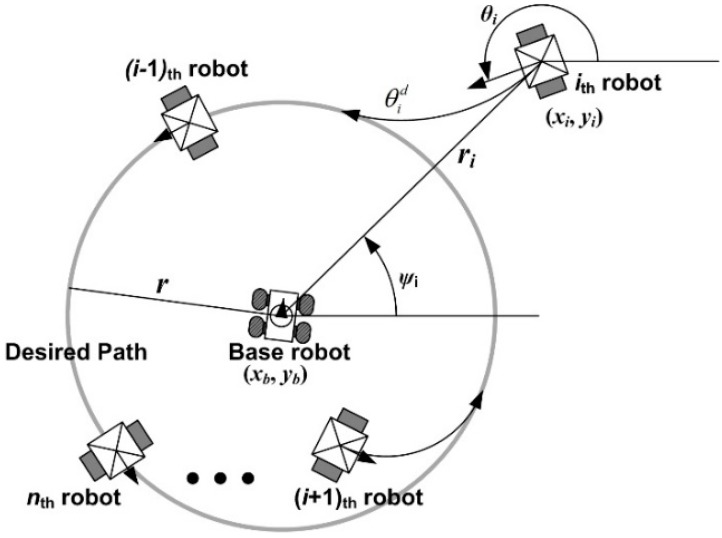
The motions of the child robots following the desired circular path.

To control child robots using the vision-based positioning system, the vector field-based multiple robot control laws in [27] are employed. Here, we design simplified control laws for the algorithm in [27]. Since all the child robots should be in the visible area, let them move on the circle whose center is the base robot, as depicted in Figure 4.

In Figure 4, (*x_b_, y_b_*) is the position of the base robot, *r* is the radius of the desired circular path with respect to the base robot, *r_i_* is the distance between the *i_th_* robot and the base robot, *ψ_i_* is the angular position, and $\theta_{i}^{d}$ is the desired orientation guiding the *i_th_* robot towards the desired path. To avoid collisions with obstacles, the radius of the desired circle, *r*, in Figure 4 is determined with respect to the obstacles around the base robot as follows:

*r* = *k_b_*min(**B***_ob_*) (3)

where 0 < *k_b_* < 1 is constant, **B***_ob_* is the set of the distances between the base robot and obstacles with respect to the angular position (the output of the commercialized LRF is similar to **B***_ob_*). The time derivatives of *r_i_* and *ψ_i_* are as follows:

(4) $${\dot{r}}_{i}=v_{i}\cos\left(\theta_{i}-\psi_{i}\right)$$

(5) $${\dot{\psi }}_{i}=\frac{v_{i}}{r_{i}}\sin\left(\theta_{i}-\psi_{i}\right)$$

Since all child robots should move on the circle, the child robots have the desired orientation:

(6) $$\theta_{i}^{d}\left(r_{i}\right)=\psi_{i}+\frac{\pi }{2}+\tan^{-1}\left(k_{d}e_{r}\right)$$

where *e_r_* = *r_i_* – *r*, *ψ_i_* = atan2(*y_i_*–*y_b_*, *x_i_*–*x_b_*), and *k_d_* > 0 is constant. Here, atan2(·) is a four-quadrant inverse tangent with the values in the interval (−π,π), and *k_d_* is a parameter controlling the ratio of the attraction angle towards the circle. The time derivative of the desired orientation in Equation (6) is as follows:

(7) $${\dot{\theta }}_{i}^{d}=\frac{v_{i}}{r_{i}}\sin\left(\theta_{i}-\psi_{i}\right)+\frac{k_{d}v_{i}\cos\left(\theta_{i}-\psi_{i}\right)}{1+{\left(k_{d}e_{r}\right)}^{2}}$$

To allocate the child robots regularly, the angular positions between the adjacent child robots should be maintained as *ψ_d_* = 2*π*/*n*. The control objective is that the errors chosen as:

(8) $$e_{i}^{\theta }=\theta_{i}^{d}-\theta_{i},\;e_{i}^{\psi }=\psi_{i-1}-\psi_{d}-\psi_{i}$$

converge to zero. The time derivatives of $e_{i}^{\theta }$ and $e_{i}^{\psi }$ are:

(9a) $${\dot{e}}_{i}^{\theta }=\frac{v_{i}}{r_{i}}\sin\left(\theta_{i}-\psi_{i}\right)+\frac{k_{d}v_{i}\cos\left(\theta_{i}-\psi_{i}\right)}{1+{\left(k_{d}e_{r}\right)}^{2}}-\omega_{i}$$

(9b) $${\dot{e}}_{i}^{\psi }=\frac{v_{i-1}}{r_{i-1}}\sin\left(\theta_{i-1}-\psi_{i-1}\right)-\frac{v_{i}}{r_{i}}\sin\left(\theta_{i}-\psi_{i}\right)$$

Then, we determined the control law of the child robots moving on the desired circle as:

(10a) $$v_{i}=\mu \left(\theta_{i}-\psi_{i}\right)\frac{r_{i}}{\sin\left(\theta_{i}-\psi_{i}\right)}\left({\dot{\psi }}_{i-1}+k_{\psi }e_{i}^{\psi }\right)$$

(10b) $$\omega_{i}={\dot{\theta }}_{i}^{d}+k_{\theta }e_{i}^{\theta }$$

where *k_ψ_* and *k_θ_* are positive constants, and:

(11) $$\mu \left(\theta_{i}-\psi_{i}\right)=1-\exp\left\{-\frac{{\left|\theta_{i}-\psi_{i}\right|}^{2}}{\sigma^{2}}\right\}$$

for when *σ* > 0 is constant. As mentioned in [27], the radial function *μ*(*θ_i_* – *ψ_i_*) makes control inputs in Equation (3) avoid divergence to infinite value in the case that *θ_i_* → *ψ_i_* and *ψ_i_* ± *π*. When the child robots use the designed control laws in Equation (10), the stability of the entire system is presented in the following theorem.

**Theorem** **1.** *When the control law in Equation (10) is employed to the child robots in Figure 4, their stability can be guaranteed in the sense that the error variables $e_{i}^{\psi }$ and $e_{i}^{\theta }$ are uniformly bounded, and the ultimate bounds can be made smaller by choosing a smaller value of σ and larger values of k_ψ_*.

**Proof of Theorem 1.** To show the ultimate boundness of $e_{i}^{\psi }$ and $e_{i}^{\theta }$, we choose the Lyapunov function candidate as follows:

(12) $$V=\frac{1}{2}\left\{{\left(e_{i}^{\psi }\right)}^{2}+{\left(e_{i}^{\theta }\right)}^{2}\right\}$$

whose time derivative is:

(13) $$\begin{array}{ll} \dot{V} & =e_{i}^{\psi }{\dot{e}}_{i}^{\psi }+e_{i}^{\theta }{\dot{e}}_{i}^{\theta }\\ \; & =e_{i}^{\psi }\left({\dot{\psi }}_{i-1}-\frac{v_{i}}{r_{i}}\sin\left(\theta_{i}-\psi_{i}\right)\right)+e_{i}^{\theta }\left({\dot{\theta }}_{i}^{d}-\omega_{i}\right) \end{array}$$

Substituting the control inputs in Equation (10) into Equations (13) and (13) becomes:

(14) $$\dot{V}=e_{i}^{\psi }\left\{1-\mu \left(\theta_{i}-\psi_{i}\right)\right\}{\dot{\psi }}_{i-1}-k_{\psi }{\left(e_{i}^{\psi }\right)}^{2}-k_{\theta }\mu \left(\theta_{i}-\psi_{i}\right){\left(e_{i}^{\theta }\right)}^{2}$$

Due to Equations (5) and (14) is revised to:

(15) $$\begin{array}{ll} \dot{V} & =-k_{\theta }{\left(e_{i}^{\theta }\right)}^{2}-k_{\psi }\mu \left(\theta_{i}-\psi_{i}\right){\left(e_{i}^{\psi }\right)}^{2}+e_{i}^{\psi }\left\{1-\mu \left(\theta_{i}-\psi_{i}\right)\right\}\left\{\frac{v_{i-1}}{r_{i-1}}\sin\left(\theta_{i-1}-\psi_{i-1}\right)\right\}\\ \; & \leq -k_{\theta }{\left(e_{i}^{\theta }\right)}^{2}-k_{\psi }\mu \left(\theta_{i}-\psi_{i}\right){\left(e_{i}^{\psi }\right)}^{2}+\frac{1}{r_{i-1}}\left|e_{i}^{\psi }\right|\left|v_{i-1}\right| \end{array}$$

From Equations (12)–(15), we ensure that $\dot{V}<0$ outside $\left\{\left|e_{i}^{\psi }\right|\leq \left|v_{i-1}\right|/\left(r_{i-1}k_{\psi }\mu \left(\theta_{i}-\psi_{i}\right)\right)\right\}$. In addition, if *σ* is small and *k_ψ_* is large, the ultimate bounds of $e_{i}^{\psi }$ become much smaller (Q.E.D.)

Here, it should be noted that, in the case that a child robot does not move on the circle, *ψ_i_* becomes the opposite side of the desired orientation, and in the case of a child robot following the desired circle, *ψ_i_* is the orthogonal direction of the desired orientation. If the child robot faces the direction of *ψ_i_*, the linear velocity becomes zero and the robot only turns towards the circle. Furthermore, because the radial function can be 1 and sin(*θ_i_* − *ψ_i_*) is not zero around *θ_i_* = *θ_d_*, the control input in Equation (10a) can be:

(16) $$v_{i}=\frac{r_{i}}{\sin\left(\theta_{i}-\psi_{i}\right)}\left({\dot{\psi }}_{i-1}+k_{\psi }e_{i}^{\psi }\right)$$

Thus, the errors, $e_{i}^{\psi }$ and $e_{i}^{\theta }$, can converge to zero in the case that *θ_i_* is close to *θ_d_.*

### 3.2. Behavior Based Multiple Robot Control Algorithm for UWB-Based Positioning System

When the base robot uses the UWB-based positioning system, the child robots can move anywhere in the boundary of the positioning system, even though they are invisible due to obstacles. Therefore, the behavior based control algorithm in [30,31] can be chosen as the control algorithm for the child robots. In this control algorithm, basic behaviors are designed and behaviors that are more complex are acquired by combining them. Three basic behaviors are used in proposed CESS as follows:

(a) Safe-wandering: The child robots wander around the base robot without collisions with adjacent child robots or obstacles.
(b) Dispersion: The adjacent child robots disperse.
(c) Aggregation: All the child robots move within the boundary of the positioning system.

In this paper, the outputs of the basic behaviors are the desired orientation [31] as in Table 1.

**Table 1:** The basic behaviors.

Behavior 1. Safe-wandering
$\theta_{d}^{v}=\{\begin{array}{cc} \theta -pi/4 & \text{left obstacle}\\ \theta +pi/4 & \text{right obstacle} \end{array}$
Behavior 2. Dispersion
$\theta_{d}^{d}=-\text{atan}2\left(e_{y}^{d},e_{x}^{d}\right)$ where $e_{x}^{d}=x_{d}-x$, $e_{y}^{d}=y_{d}-y$, $\left(x_{d},y_{d}\right)$ is the mean position of the two nearest robots
Behavior 3. Aggregation
$\theta_{d}^{a}=\text{atan}2\left(e_{y}^{a},e_{x}^{a}\right)$ where $e_{x}^{a}=x_{a}-x$, $e_{y}^{a}=y_{a}-y$, $\left(x_{a},y_{a}\right)$ is the base robot

**Figure 5:**
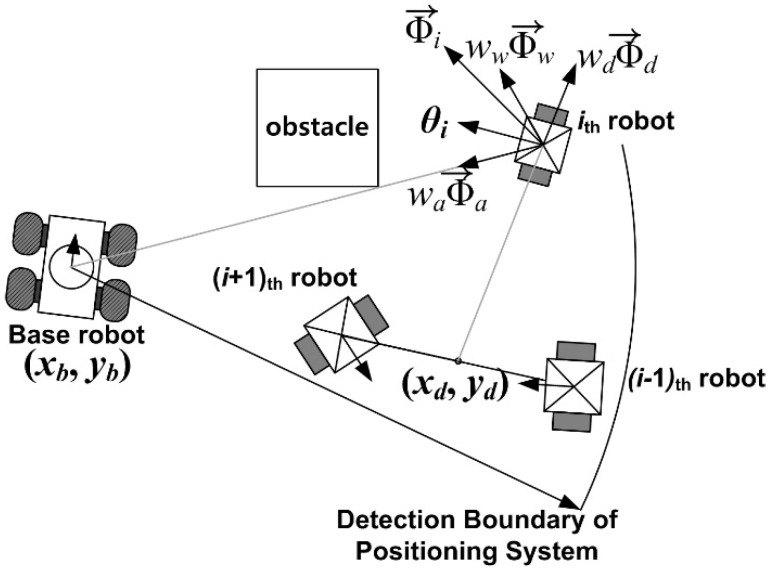
The combination of the basic behaviors.

If the basic behaviors are described as a normalized vector with respect to their outputs, the combined behavior of the child robots is acquired by linear weighted vector summation, as in Figure 5.

**Figure 6:**
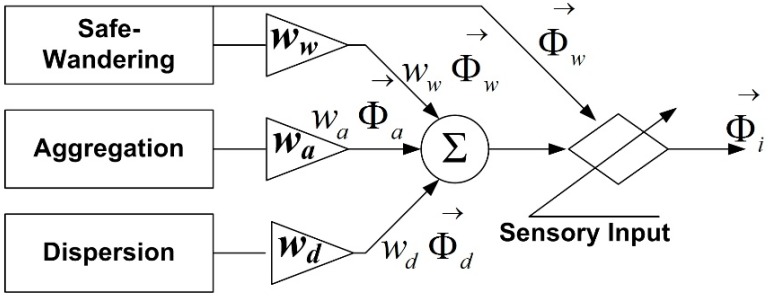
The combination of the basic behaviors.

Figure 5 shows the combination of the three basic behaviors as in (17):

(17) $$\vec{\Phi_{i}}=w_{a}\vec{\Phi_{a}}+w_{d}\vec{\Phi_{d}}+w_{w}\vec{\Phi_{w}}$$

where $\vec{\Phi }$ is the normalized vector, *w* is the weighting factor, and *i*, *a*, *d*, and *w* mean the outputs of the *i_th_* robot, aggregation, dispersion, and safe-wandering, respectively. Accordingly, the desired orientation of the *i_th_* robot can be chosen as a direction of $\vec{\Phi_{i}}$, as depicted in Figure 6.

As can be seen in Figure 6, the basic behaviors are combined by the weighted vector summation, and the collision avoidance using the safe-wandering behavior is included to avoid collisions. To control the child robots, the linear velocity of all the robots is chosen as a constant value, *v_i_* = *v_d_*, and the angular velocity should be designed to achieve the desired orientation, which is the angle of $\vec{\Phi_{i}}$.

## 4. Simulation Results

To show the performance of CESS, numerical simulations with four scenarios are included. In these scenarios, the base robot has twelve child robots, which have two one-beam range finders with 4 m range and 0.01 m resolution. Because the objective of the proposed CESS is the replacement of LRF, all scenarios are compared with the numerical models of URG-04LX by Hokuyo [32], which is a 1-ch LRF, and the HDL-32E by Velodyne [33], which is a 32-ch LRF.

**Figure 7:**
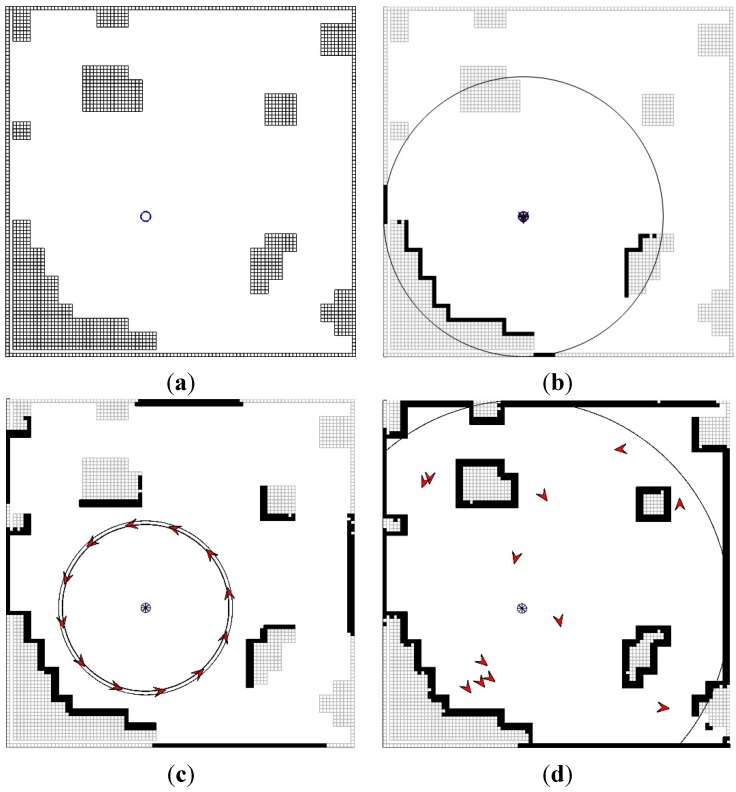
The performance of CESS in the 2D plane. (**a**) The obstacle map; (**b**) The detected obstacles by LRF; (**c**) The detected obstacles by CESS, based on the vision-based positioning system; (**d**) The detected obstacles by CESS based on the UWB-based positioning system.

The first and second scenarios show the performance of CESS replacing the 1-ch LRF using the vision- and UWB-based positioning systems, respectively. The work space is 10 m × 10 m, and the initial conditions of the base and child robots are determined as follows: (*x_b_*(0), *y_b_*(0), *θ_b_*(0)) = (4, 4, −*π*/2) and (*x_i_*(0), *y_i_*(0), *θ_i_*(0)) = (*x_b_*(0) + 2.5cos(2*πi/*12), *y_b_*(0) + 2.5sin(2*πi/*12), 2*πi/*12 + *π*/2). In addition, the obstacle information is marked on the grid map where each grid is 0.1 m × 0.1 m. To compare with commercialized LRF, a virtual URG-04LX is used, which has the following specifications: a distance range of 4 m, an angular range of −120°–120°, and 0.001 m resolution. Figure 7 shows the results of the first and second scenarios.

Figure 7a shows the given unknown environment. Figure 7b shows the performance of the LRF. The big circle in Figure 7b shows the boundary of the LRF. Figure 7c,d show the performance of the CESS using the vision- and UWB-based positioning systems, respectively. In Figure 7c, the big circles show the changes of the desired circle of the child robots that result from Equation (3), which makes the child robot move in the visible area of the base robot without colliding with obstacles. In Figure 7d, the big circle shows the boundary of the UWB-based positioning system on the base robot. From Figure 7b–d, LRF provides exact information about the obstacles in the limited area; meanwhile, it can be ensured that the proposed CESS system extends the detectable area. In particular, as in Figure 7d, in the case of the UWB-based positioning system, it is possible that the hidden obstacles are detected. In addition, in Figure 7c,d, there are position errors for the obstacles, because of the quantization errors derived from marking the obstacle information on the grid map. However, as can be seen in Figure 7c,d, the marking grids are similar to the real map.

**Figure 8:**
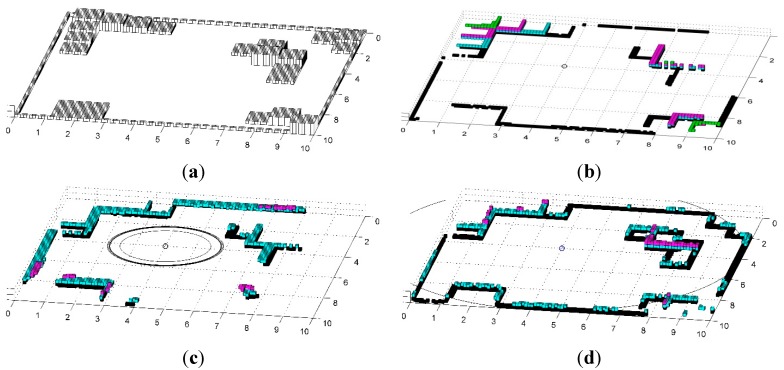
The performance of CESS in a 3D space. (**a**) The obstacle map; (**b**) The detected obstacles by LRF; (**c**) The detected obstacles by CESS, based on the vision-based positioning system; (**d**) The detected obstacles by CESS based on the UWB-based positioning system.

The third and fourth scenarios show the performance of obstacle detection in 3D space, as in Figure 8 where the following cases are included: (a) Virtual HDL-32E with 100 m range and 0.001 m resolution; (b) The child robots with two one-beam distance finders whose vertical angles are *δ_i_* = 2*π* (*i* − 1)/180 for *i* = 1, …, *n* and range is 4 m using the vision-based positioning system, and (c) the child robots using the UWB-based positioning system whose range is 6 m. In these scenarios, the initial conditions of the base and child robots are determined as follows: (*x_b_*(0), *y_b_*(0), *θ_b_*(0)) = (4, 4, *π*/4) and (*x_i_*(0), *y_i_*(0), *θ_i_*(0)) = (*x_b_*(0) + 1.5cos(2*πi/*12), *y_b_*(0) + 1.5sin(2*πi/*12), 2*πi/*12 + *π*/2). In addition, the obstacle information acquired via child robots will be marked on the grid map where each grid is 0.1 m × 0.1 m × 0.1 m.

The given environment is depicted in Figure 8a. Figure 8b shows the results of the 32-ch LRF, such that, the obstacles are described exactly in the large area since the virtual LRF has the distance range of 100 m. Meanwhile, Figures 8c,d show the results of CESS using the vision- and UWB-based positioning systems, respectively. While the child robots have range finders with a short range, they can cover a large area, because the detectable area is extended by the positioning systems on the base robot. In particular, the proposed system using the UWB-based positioning system presented in Figure 8d can acquire information about obstacles hidden behind others.

**Remark** **2.** *In the scenarios, the resolution of the range finders on the child robots is 0.01 m. Contrasting with LRFs whose resolutions are 0.001 m, the accuracy of the range finders on the child robot are under 10% of the LRF. Despite the big performance difference between LRF and the range finders on the child robots, the proposed CESS shows similar performance to LRF and the extension of the detectable area, because CESS with multiple child robots provides information using a number of robots instead of a single device*.

From the results of the four scenarios, it can be ensured that the proposed CESS provides similar performance to high-cost LRF, extends the detectable area, and acquires information in the invisible area that cannot be measured by LRF. In addition, the scenarios show that the sensitivity of position errors derived from measurement and communication noises and communication delay are reduced because of the quantization errors included in the grid map.

## 5. Conclusions

We proposed CESS with multiple child robots that can be utilized instead of LRF. Unlike the robot system with high-performance LRF, which has a high cost, the proposed CESS acquires obstacle information using multiple child robots with low performance range finders, which have a low cost. To control multiple child robots, the vector field–based multiple robot control algorithm using the vision-based positioning system, and the behavior-based control algorithm using the UWB-based positioning system are employed. From these positioning systems, certain advantages that surpass the performance of LRF, such as the extension of the scanning range and the capability to detect hidden obstacles are achieved. The simulation results are included to show the contributions and performance of the proposed cooperative environment scan system by contrasting it with LRF. In future research, for real robots, the acquisition of robustness against the communication delay resulting from increased child robots (*i.e.*, scalability) will be pursued, and these will be implemented to actual robot system.

## References

[1] Latombe J.-C. (1991). Robot Motion Planning.

[2] Lavalle S. (2006). Planning Algorithms.

[3] Yang D.-H. (2006). A Collision Avoidance Algorithm for Multiple Mobile Robots Using Roadmaps. Ph.D. Thesis.

[4] Thrun S., Burgard W., Fox D. (2005). Probabilistic Robotics.

[5] Rekleitis I.M. (2004). A Particle Filter Tutorial for Mobile Robot Localization.

[6] Durrant-Whyte H., Bailey T. (2006). Simultaneous localization and mapping (SLAM): Part I the essential algorithms. IEEE Robot. Autom. Mag..

[7] Durrant-Whyte H., Bailey T. (2006). Simultaneous localization and mapping (SLAM): Part II state of the art. IEEE Robot. Autom. Mag..

[8] Do Y., Kim J. (2013). Infrared range sensor array for 3D sensing in robotic applications. Int. J. Adv. Robot. Syst..

[9] Jiménez F., Naranjo J.E., Gómez O., Anaya J.J. (2014). Vehicle tracking for an evasive manoeuvers assistant using low-cost ultrasonic sensors. Sensors.

[10] Schwarz B. (2010). LIDAR: Mapping the world in 3D. Nat. Photonics.

[11] Pandey G., McBride J.R., Eustice R.M. (2011). Ford campus vision and lidar data set. Int. J. Robot. Res..

[12] Lacaze1 A., Murphy M., Giorno M.D., Corley K. (2012). Reconnaissance and autonomy for small robots (RASR) team: MAGIC 2010 Challenge. J. Field Robot..

[13] Butzke J., Daniilidis K., Kushleyev A., Lee D.D., Likhachev M., Phillips C., Phillips C. (2012). The University of Pennsylvania MAGIC 2010 multi-robot unmanned vehicle system. J. Field Robot..

[14] Foix S., Alenyà G., Torras C. (2011). Lock-in time-of-flight (ToF) cameras: A survey. IEEE Sens. J..

[15] May S., Fuchs S., Droeschel D., Holz D., Nüchter A. Robust 3D-mapping with time-of-flight cameras. Proceedings of the International Conference on Intelligent Robots and Systems.

[16] Howard A. (2006). Multi-robot simultaneous localization and mapping using particle filters. Int. J. Robot. Res..

[17] Mourikis A.I, Ourmeliotis S.I. (2006). Predicting the performance of cooperative simultaneous localization and mapping (C-SLAM). Int. J. Robot. Res..

[18] Lee H.-C., Lee S.-H., Lee T.-S., Kim D.-J., Lee B.-H. A Survey of map merging techniques for cooperative-SLAM. Proceedings of the International Conference on Ubiquitous Robots and Ambient Intelligence.

[19] Howard A., Parker L.E., Sukhatme G. (2006). Experiments with a large heterogeneous mobile robot team: Exploration, mapping, deployment, and detection. Int. J. Robot. Res..

[20] Cruz D., McClintock J., Perteet B., Orqueda O.A.A., Cao Y., Fierro R. (2007). Decentralized cooperative control: A multivehicle platform for research in networked embedded systems. IEEE Control Syst. Mag..

[21] Kim J.H., Kwon J.-W., Seo J. (2014). Multi-UAV-based stereo vision system without GPS for ground obstacle mapping to assist path planning of UGV. Electron. Lett..

[22] Das A.K., Fierro R., Kumar V., Ostrowski J.P., Spletzer J., Taylor C.J. (2002). A vision-based formation control framework. IEEE Trans. Robot. Autom..

[23] Fontana R. Advances in ultra wideband indoor geolocation systems. Proceedings of the 3rd IEEE Workshops on WLAN.

[24] Fontana R. (2004). Recent system applications of short-pulse ultra-wideband (UWB) technology. IEEE Trans. Microw. Theory Tech..

[25] Pahlavan K., Li X., Makela J.-P. (2002). Indoor geolocation science and technology. IEEE Commun. Mag..

[26] Kwon J.-W., Park M.-S., Chwa D. Localization of the mobile agent using indirect Kalman filter in distributed sensor networks. Proceedings of the International Conference on Ubiquitous Information Management and Communication.

[27] Chwa D. (2004). Sliding-mode tracking control of nonholonomic wheeled mobile robots in polar coordinates. IEEE Trans. Control Syst. Technol..

[28] Kwon J.-W., Chwa D. (2012). Hierarchical formation control based on a vector field method for wheeled mobile robots. IEEE Trans. Robot..

[29] Ghabcheloo R., Pascoal A., Silvestre C., Kaminer I. (2005). Coordinated path following control of multiple wheeled robots linearization techniques. Int. J. Syst. Sci..

[30] Mataric M.J. (1994). Interaction and Intelligent Behaviors. Ph.D. Thesis.

[31] Kwon J.-W., Kim J.H., Seo J. Consensus-based obstacle avoidance for robotic swarm system with behavior-based control scheme. Proceedings of the International Conference on Control, Automation and Systems 2014.

[32] Scanning Range Finder (SOKUIKI Sensor), HOKUYO. http://www.hokuyo-aut.jp/02sensor/07scanner/urg_04lx.html.

[33] HDL-32E, Velodyne. http://velodynelidar.com/lidar/hdlproducts/hdl32e.aspx.

